# Analysis of the work environment and intention of perioperative nurses to quit work*

**DOI:** 10.1590/1518-8345.3239.3256

**Published:** 2020-04-17

**Authors:** Amalia Sillero-Sillero, Adelaida Zabalegui

**Affiliations:** 1 Hospital de la Santa Creu i Sant Pau, Perioperative Nursing, Barcelona, Spain.; 2 Hospital Clínic, Deputy of Director of Nursing Research and Education. Barcelona, Spain.

**Keywords:** Nurse Turnover, Health Facility Environment, Perioperative Nursing, Job Satisfaction, Burnout, Professional, Retention, Rotatividade de Enfermeiros, Ambiente de Instituições de Saúde, Enfermagem Perioperatória, Satisfação no Emprego, Esgotamento Profissional, Retenção, Rotación Enfermera, Ambiente de Instituciones de Salud, Enfermería Perioperatoria, Satisfacción en el Trabajo, Agotamiento Profesional, Retención

## Abstract

**Objective:**

to investigate how the perioperative work environment affects work dissatisfaction, professional exhaustion and the perception of the quality of care about the intention of abandoning the work of perioperative nurses.

**Method:**

cross-sectional study with 130 nurses working in the surgical area of a high-tech Spanish public university hospital. The scale of the nursing practice environment, Maslach’s exhaustion inventory, the questions about job satisfaction, the perception of the care quality and intention to abandon work to collect data were used. Descriptive, inferential and logistic regression statistics were made.

**Results:**

in general, 20% of perioperative nurses would want to quit their work. The dimension of the work environment of staff and resources, dissatisfaction and emotional exhaustion in nurses were factors that indicated the intention of perioperative nurses to abandon work.

**Conclusion:**

the implementation of strategies for the retention of perioperative nurses should be considered, improving the factors that indicate how the work environment, especially the allocation of personnel and resources, dissatisfaction and emotional exhaustion. Creating positive work environments based on magnetic values can be a key strategy.

## Abstract (pt)

**Objetivo:**

investigar como o ambiente laboral perioperatório afeta a insatisfação laboral, a exaustão profissional e a percepção da qualidade do cuidado sobre a intenção de abandonar o trabalho, das enfermeiras perioperatórias.

**Método:**

estudo transversal com 130 enfermeiras que trabalham na área cirúrgica de um hospital universitário público espanhol de alta tecnologia. Foi utilizada a escala do ambiente da prática de enfermagem, o inventário de burnout de Maslach, as perguntas sobre a satisfação laboral, a percepção da qualidade do cuidado e a intenção de abandonar o trabalho, para a coleta de dados. Foram realizadas estatísticas descritivas, inferenciais e de regressão logística.

**Resultados:**

em geral, 20% das enfermeiras perioperatórias gostariam de abandonar o trabalho. A dimensão do ambiente laboral em relação aos investimentos em pessoal e recursos, a insatisfação e a exaustão emocional das enfermeiras foram os fatores preditores que indicaram a sua intenção de abandonar o trabalho.

**Conclusão:**

deve-se considerar implementar estratégias de retenção das enfermeiras perioperatórias, melhorando os fatores preditores que indicam, como o ambiente laboral, especialmente a dotação de pessoal e os recursos, a insatisfação e o esgotamento emocional. Criar ambientes de trabalho positivos, baseados nos valores magnéticos, pode ser uma estratégia-chave.

## Abstract (es)

**Objetivo:**

investigar cómo el entorno de trabajo perioperatorio afecta la insatisfacción laboral, el agotamiento profesional y la percepción de la calidad del cuidado, sobre la intención de abandonar el trabajo de las enfermeras perioperatorias.

**Método:**

estudio transversal con 130 enfermeras que trabajan en el área quirúrgica de un hospital universitario público español de alta tecnología. Se utilizó la escala del entorno de la práctica enfermera, el inventario de agotamiento de Maslach, las preguntas sobre la satisfacción laboral, la percepción de la calidad del cuidado e intención de abandonar el trabajo para recoger datos. Se hicieron estadísticas descriptivas, inferenciales y de regresión logística.

**Resultados:**

en general, el 20% de las enfermeras perioperatorias desearían abandonar su trabajo. La dimensión del entorno de trabajo de dotación de personal y recursos, la insatisfacción y el agotamiento emocional en las enfermeras fueron factores predictores de la intención de las enfermeras perioperatorias de abandonar el trabajo.

**Conclusiones:**

Se ha de considerar implementar estrategias de retención de las enfermeras perioperatorias, mejorando los factores predictores como el entorno laboral, especialmente la dotación de personal y recursos, la insatisfacción y el agotamiento emocional. Crear entornos de trabajo positivos, basados en los valores magnéticos, puede ser una estrategia clave.

## Introduction

The lack of nursing professionals is well documented and it is an important concern of health systems due to its effects on the quality of patient care, nurse satisfaction and financial burden in health services^(^ 1 ^-^ 2 ^)^. In particular, a growing lack and turnover of perioperative nurses is expected, although it is expected that 20% of the people who are employed in this specialty will retire in the coming years^(^ 3 ^)^. The demand for perioperative nurses has already been increasing, growing about 1% to 2% each year, as the volume of surgeries increases due to an aging population and to demands for greater care, as well as by more complex interventions^(^ 4 ^)^.

Moreover, the recruitment and retention of nurses is difficult due to the conditions of the perioperative environment, where nurses are required to have the specific skills to work with a multidisciplinary team, in an environment complex, high-tech, and with high patient turnover^(^ 5 ^)^; therefore, solutions must be sought to face such staff relay, to retain nurses and to decrease the turnover in the perioperative environment.

Study show that the worst working environments are related to increased burnout^(^ 6 ^)^, lower job satisfaction and a greater intention to quit work^(^ 7 ^)^ and that better environments have less burnout, greater job satisfaction and lower intention to abandon^(^ 8 ^)^.

Most studies have measured the perceptions of nurses working in different acute care units and that there is little understanding of the factors of the work environment that are related to labor dissatisfaction and the intentions of nurses perioperative to abandon. In the United States (USA), one study examined 11 units with different work environments and the researchers showed that, among them, perioperative environments were the least favorable^(^ 9 ^)^. Therefore, the relationships between the work environment, labor dissatisfaction, professional exhaustion and the intentions of perioperative nurses to abandon work must be investigated. In a national study that examined the nurses’ work satisfaction, using a sample of 5654 in 59 hospitals in Spain, the researchers found that nurses’ work satisfaction varied according to the work unit and that 26% of them would want to leave their hospital as a consequence of unfavorable work environments, job dissatisfaction and high levels of burnout^(^ 10 ^)^. Nevertheless, this study was not conducted in perioperative units and, although there are studies in other environments on related factors such as emotional exhaustion, work overload and nurses’ job dissatisfaction with the intentions of abandoning work^(^ 11 ^-^ 12 ^)^. The study in perioperative environments are very scarce^(^ 13 ^)^, which indicates the need to investigate the factors related to the perioperative environment to develop optimal ways to relieve the lack and turnover of nurses.

One of the measures proposed in the international literature offers an organization model with adequate staff allocation, lower workloads and work support environments is related to Magnetic Recognition. For three decades, the development of *Magnetic Hospitals* was one of the effective strategies to solve the lack of nursing professionals. Since its inception in the USA in the 1990s, it has expanded to different types of service centers to different countries and changed its name by the Magnetic Recognition Program of the American Nurse Credentialing Center (ANCC)^(^ 14 ^)^. Magnetic Hospitals have become a standard for the excellence of nursing care and the work environment. Nurses have lower turnover, greater job satisfaction, autonomy in the environment of professional practice and good interdisciplinary work relationships. Magnetic Recognition refers to complying with five values that bring together the 14 forces of magnetism (FOM) and form the creation of high quality nursing work environments^(^ 15 ^)^, they are: 1- Implementation of a transformative leadership (2 forces: the quality of nursing leadership and management style; 2 - Development of an empowered structure (5 forces: organizational structure, policies and personnel programs, the community and health organization, the nursing image and professional development); 3 - Establishment of good practices (5 forces: professional models of care, consultation and resources, autonomy, teachers and interdisciplinary relationships); 4 - Use of knowledge, innovation, improvement continues through research, evidence-based practice and innovation (1 strength: quality improvement) and 5 - Care outcomes and identify the factors that influence the results of patients and professionals^(^ 16 ^)^.

Studies on the lack of perioperative nurses and the implementation of initiatives in Spain to improve it remains limited^(^ 17 ^)^. Therefore, taking the previous study as the reference, the research question was: how does it affect the environment of perioperative nursing practice in job satisfaction, professional exhaustion, and the intention to abandon work? From there, the aim of this study was to investigate how the perioperative work environment affects work dissatisfaction, professional exhaustion and the perception of the quality of care about the intention of abandoning the work of perioperative nurses.

## Method

This was cross-sectional study. Data were collected in 2014-15 and no sample calculation, although all nurses working in the surgical area of a high-tech public university hospital in Spain were included. The inclusion criteria were work experience greater than 1 year in the place of work and being active during the data collection period, for N=130 nurses from the population that was studied.

The key variables of this study were the work environment, burnout level, perception of the quality of care provided, intention to abandon work and job satisfaction. To measure the work environment, one of the most used instruments worldwide was used to determine the quality of the practice environments, the PES-NWI (Practice Environment Scale of the Nursing Work Index)^(^ 18 ^)^ and which was validated in Spanish^(^ 19 ^)^. The scale consists of five dimensions with 31 items that describe a set of characteristics that are present in organizations that support the practice of professional nursing. Nurses thus indicate the degree, according to what is present in each item in their work. The five subscales or dimensions comprising PES-NWI are: 1) Allocation of adequate personnel and resources; 2) Leadership skills and support from those responsible; 3) Nurse-physician relationships; 4) Nurses’ foundations for quality and 5) Participation of nurses in hospital matters. Reliability (i.e., Cronbach’s alpha coefficients) was 0.90 (95%CI: 0.87-0.93)^(^ 19 ^)^. Regarding the PES-NWI responses, the mean of each dimension was performed. The overall PES-NWI score was the average of subscale or dimension scores. To describe and compare magnetic characteristics in the perioperative unit with the results of the international research on *Magnetic Hospitals*, the dimensions of the PES-NWI and the overall scores at the hospital level were added. For each dimension, the mean scores ranged from 1 to 4, with the value of 2.5 as neutral midpoint (i.e., neither agreement nor disagreement). Values greater than 2.5 indicate that PES-NWI elements are present in the current work environment; values below 2.5 indicate disagreement. The environment is classified as favorable, if four or five average scales of dimensions are greater than 2.5; as mixed, if two or three dimensions had average scores greater than 2.5 and as unfavorable, if neither or one of the five subscales or dimensions achieved an average score of 2.5. For this study, Cronbach reliability or α for dimensions ranged from 0.86 to 0.91 and for the score composed of the entire Cronbach α scale 0.89.

The exhaustion of the nurse at work was measured using the Maslach Burnout Inventory (MBI)^(^ 20 ^)^, in its version validated to the Spanish language^(^ 21 ^)^. The instruments consists of 3 dimensions: Emotional exhaustion (EE), Depersonalization (DP) and Personal Achievement (PA), including 22 items measured on a Likert scale of 1 to 6 points (‘never’, up to ‘every day’). The EE contains 9 items; the DP, 5 items and PA, 8 items with a maximum score of 54.48 and 30, respectively. They were categorized into 3 groups each (low, medium and high), according to the values: EE: low ≤ 18, mean [19-26], high ≥ 27; DP: low ≤ 5, medium [6-9], high ≥ 10; PA: low ≥ 40, medium [39-34], high ≤ 33. For global valuation, High Burnout occurred when 2 to 3 dimensions present high levels; when 2 or more dimensions have average or well one scale valuations at each level and low, when more than two dimensions have low levels, following the criteria that are used in the RN4Cast Spain study^(^ 10 ^)^. Following the methodology used by the study of the European RN4Cast^(^ 22 ^)^, work satisfaction (WS) was evaluated using nine articles that point out the specific aspects of job satisfaction: flexible schedules, professional development, autonomy, status, salary, training, vacations, dismissals and licenses for studies. All items used a 4-point Likert scale, ranging from 1, “very dissatisfied” to 4, “very satisfied”. In addition, the perception of the quality of care was established by one question: “In general, how would you describe the quality of nursing care that is delivered to patients in your unit?”. They could respond to this question on a four-point response scale with ‘bad’, ‘regular’, ‘good’ or ‘excellent’. Finally, the intention to abandon the work that was measured by a single binary response (yes/no). The measurement instruments that have been used are valid and reliable in the context of study. The Research Ethics Committee of the hospital (CEIC Code: 47/2013) approved this study and all participants provided their informed consent.

A descriptive analysis of all variables was performed using mean, standard deviation and minimum and maximum values, according to the distribution of quantitative variables, frequencies, and percentages for qualitative variables. Initially, a normality test using the normal Q-Q graph and the Kolmogorov-Smirnov goodness adjustment test were performed for correlations. PES-NWI variables admit the normal model. Nevertheless, the satisfaction variables are far from the normal model, which is why non-parametric tests were used. Pearson and Spearman’s correlations were used, according to the variables, to examine the strength and direction of bivariate relationships between the key variables of the study. Finally, a multivariate logistic regression analysis was performed. Statistical significance was set at p < 0.05. The statistical software SPSS V22 was used.

This study also compared the PES-NWI values of perioperative nurses with the values of *Magnetic Hospitals* ^(^ 23 ^)^ and the hospitals of the RN4CAST Spain^(^ 10 ^)^ study, obtained from the literature review in different databases (Pubmed, CINAHL, Scopus). The mean score per item, subscale mean or dimension, global mean, and levels are reported.

## Results

In total, 130 questionnaires were obtained that were completed and the overall response rate was 98%. Most were women (91.5%, n = 119) and married (75%, n = 57). The mean age was 44.5 years (SD = 11.90); mean professional experience in the institution was 20 years (SD = 12.3) and in the unit, 13.9 years (SD = 11.14). Most professionals (73%, n = 96), worked in the operating room (83%, n = 108), had a fixed contract (58.5%, n = 76), and worked in the morning shift. Regarding the academic degree, workers presented undergraduate degrees in nursing (13.8%, n = 18), master’s degree (31.5%, n=41), postgraduate degree in surgical nursing (67%, n=67) and none had a doctoral title. Regarding continued training, 48.5%( n=63) of the nurses studied < 50 h over the last 24 months and 17% (22) studied > 300 h. The mean number of hours worked in the last shift of the hospital was 8.4 h and 26.2% (n = 34) of the nurses stated that they had worked more hours than what is established in their contracts.

Characteristics of the work environment: nurses classified the nursing fundamentals for quality (M = 2.65, SD=0.52; range 2.56-2.74) as the most favorable characteristic present in the work environment, followed by nurse-physician relationships (M = 2.47 SD = 0.68; range 2.35-2.59). The skill, leadership and support of those responsible (M = 2.27, SD= 0.67; range 2.15-2.39) and the suitability of personnel and the resources (M = 2.12, SD = 0.65; range 2.01-2.23) did not receive favorable evaluations (score < 2.5) and, finally, the participation of nurses in hospital affairs (M =1.96, SD = 0.43; range 1.88-2.03) was qualified as the least favorable characteristic of this environment. Therefore, the findings showed an unfavorable perioperative work environment, although there was only one subscale that had an average score greater than 2.5. The data that were obtained from PES-NWI provided information regarding magnetic characteristics. The highest points were: we work with clinically competent nurses (M = 3.51, SD = 0.65), followed by care are based on a nursing model (M = 3.16, SD = 0.91) and administrators expect a high level of care (M = 2.89, SD = 1.18) belonging to the dimension of nurse fundamentals for quality care. While the least present elements are the opportunities to participate in hospital decisions (M = 1.33, SD = 0.62), an administration that listens and responds to employee concerns (M=1.56, SD = 0.73) and opportunities to ascend within the organization (M =1.65, SD = 0.70). They all belong to the nurse’s participation dimension in hospital affairs. The results are presented in Table 1.

**Table 1:** Descriptive analysis of the subscales or dimensions of PES-NWI. (n=130 perioperative nurses). Barcelona, Spain, 2014-2015

Subscales-*Dimensions PES-NWI* *		M^†^ DE^‡^	Range
*1: Staff allocation and adequate resources*	2.12 (0.65)	2.01	2.23
There are adequate support services	2.67 (1.02)	2.49	2.85
There is enough time and opportunities to expose care problems to other nurses	1.94 (0.84)	1.79	2.08
There are enough nurses to provide care	1.95 (0.85)	1.81	2.10
There is enough staff to do the work	1.92 (0.81)	1.78	2.06
*2: Nurse-physician work relationship*	2.47 (0.68)	2.35	2.59
Physicians and nurses have a good working relationship	2.75 (0.67)	2.64	2.87
There is a lot of teamwork among medical nurses	2.35 (0.83)	2.21	2.50
There is (joint) collaboration between medical nurses	2.30 (0.89)	2.15	2.45
*3: Skill, leadership and support of your guardians*	2.27 (0.67)	2.15	2.39
Supervisors support from physicians	2.42 (0.81)	2.28	2.56
The supervisor is a good manager and leader	2.40 (0.89)	2.24	2.56
If they make compliments-recognitions to the work well done	1.92 (0.82)	1.77	2.06
The supervisor supports the decisions of the staff	2.39 (0.87)	2.24	2.54
Supervisors use the errors to learn	2.22 (0.88)	2.07	2.38
*4: Nursing fundamentals and quality care*	2.65 (0.52)	2.56	2.74
There is continued training for nurses	2.57 (0.93)	2.41	2.73
Management expects high quality nursing care	2.89 (1.18)	2.69	3.10
There is clear philosophy of nursing environment for the patient	2.75 (0.89)	2.60	2.91
If you work with clinically competent nurses	3.51 (0.65)	3.39	3.62
There is an active quality assurance program	2.78 (0.94)	2.62	2.95
There is a guidance program for new nurses	1.77 (0.90)	1.61	1.93
Nurse care is based on a nurse model	3.16 (0.91)	3.00	3.32
Carry out patient care and writing plans	2.59 (1.04)	2.41	2.77
If you plan care to foster your continuity	2.25 (1.06)	2.06	2.43
Nursing diagnoses are used	2.25 (1.04)	2.07	2.43
*5: Participation of nurses in hospital matters*	1.96 (0.43)	1.88	2.03
There are promotion opportunities at the professional level	1.88 (0.87)	1.73	2.04
There are opportunities in decisions on hospital management	1.33 (0.62)	1.22	1.44
The /A director of nursing is very visible and accessible	1.73 (0.90)	1.57	1.89
Authority nurse direction as other directions.	2.18 (0.94)	2.01	2.34
There are opportunities to ascend	1.65 (0.70)	1.53	1.78
The direction listens and responds to concerns	1.56 (0.73)	1.44	1.69
Nurses get involved in internal management.	2.32 (0.84)	2.18	2.47
Nurses have the opportunity to form the committees	2.75 (0.86)	2.60	2.89
The supervisor consults the staff of daily problems	2.20 (0.93)	2.04	2.36

* PES-NWI = Practice Scale-Nursing Work Index;

† M = Mean;

‡ SD = Standard Deviation.

Regarding the comparisons of PES-NWI values, it was found that the levels of the scores of the global PES-NWI scale of the perioperative unit was lower (range 2.20-2.40) than the score of Magnetic Hospitals (range 2.92-3.00). The difference to the hospitals of the study of Spanish hospitals was small (range 2.27-2.42). For the five dimensions, the levels of the perioperative unit were lower than those of Magnetic Hospitals. However, the levels of the PES-NWI subscales in the perioperative unit as “nursing fundamentals for the quality of care” and “nurse-physician relationships” were higher than the levels of those studied in Spain. In general, the scores that were obtained in this study indicate that nurses have similar qualifications of the work environment as Spanish hospitals and lower than those obtained in Magnetic Hospitals. The results are shown in Table 2

**Table 2:** Mean levels of PES-NWI: perioperative unit. Barcelona, Spain, 2014-15; Hospitals study. Spain, 2012; Magnetic hospitals, USA, 2014

PES-NWI* Dimensions		Mid-level		
		Perioperative unit Spanish 2014-2015	RN4Cast^†^ Hospitals Spain 2012	*Magnetic hospitals* USA^‡^ 2014
*1*	*Allocation of adequate personnel and resources*	2.01 - 2.23	2.44 - 2.56	2.65 - 2.88
*2*	*Collegiate relationships between nurse and physician*	2.35 - 2,59	1.99 - 2.08	2.99 - 3.07
*3*	*Skill, Leadership and Support of those responsible*	2.15 - 2.39	2.42 - 2.61	2.72 - 3.07
*4*	*Nurse fundamentals for the quality of care*	2.56 - 2.74	2.37 - 2.45	3.09 - 3.20
*5*	*Nurse participation in hospital affairs*	1.88 - 2.03	2.14 - 2.41	2.76 - 3.01
*Global*		2.20 - 2.40	2.27 - 2.42	2.92 - 3.00

* PES-NWI = Practice Scale-Nursing Work Index;

† RN4Cast Spain (Forecasting Register Nurse in Spain);

‡ USA = United States

Regarding burnout, perioperative nurses presented moderate levels (global burnout: mean 47%, n=36 and high level 6%, n=5. High levels of burnout were identified in 25% (n=19) of nurses had high levels of exhaustion. High levels of depersonalization were observed in 10% (n=8) of the sample, and high levels of personal achievement in 21% (n=16).

Regarding job satisfaction, in all items, the highest response percentage was not dissatisfied, always above 52% and even reaching 98%. Despite the minimum differences between one and other variables, the highest mean, which indicates the highest degree of satisfaction, was observed in the item flexible hours. On the other hand, the variables with the highest degree of dissatisfaction were salaries, the opportunity for professional development and the licenses for studies. In total, 74.3% (n=97) of the nurses were little dissatisfied with their current work in the hospital, and 20% (n=26) intended to leave their position. Regarding the quality of care in their unit, more than half of those who were interviewed reported good quality care 61% (n=79) and excellent 14% (n=11). Results are shown in Table 3.

**Table 3:** Descriptions of burnout results, labor satisfaction, intention to abandon and perception quality of care (n=130). Barcelona, Spain, 2014-2015

***Emotional exhaustion***	N	%			
Low level	74	56.9			
Mid-level	31	24			
High level	25	19			
Depersonalization					
Low level	102	78			
Mid-level	18	14			
High level	10	7.7			
Personal achievement					
Low level	61	46			
Mid-level	48	37			
High level	21	16			
Burnout Global					
Low level	77	59.2			
Mid-level	47	36.2			
High level	6	4.6			
**Satisfaction**	**Very Dissatisfied**		**Dissatisfied**	**Moderately Satisfied**	**Very Satisfied**
Job satisfaction	3.7 %		74.3 %	22.0 %	0.0 %
Hourly flexibility	2.9 %		52.3 %	34.9 %	10.0 %
Opportunity	2.8%		92.1%	5.1%	0.0 %
Autonomy	5.1 %		77.4 %	9.7 %	7.8 %
Wages	1.3 %		94.6 %	4.1 %	0.0 %
Training	10.7 %		57.9 %	21.6 %	9.8 %
Vacation	1.7 %		78.1 %	20.3 %	0.0 %
Low illness	1.3 %		91.0 %	7.7 %	0.0 %
License for studies	0.0 %		97.9 %	2.1 %	0.0 %
Intention to abandon	Yes 20% (26)			No 80% (110)	
*Quality of Care*	Bad 5% (6)		Regular 24% (30)	Good 61% (79)	Excellent 14% (11)

In the correlations between the dimensions of PES-NWI and the other independent variables, it was found that most correlations do not reach statistical significance (p>0.05) and have low intensity values. It was only discovered that job satisfaction and allocation of resources and personnel (r=0.013), and emotional exhaustion (r=-0.206), are significantly associated. Table 4 presents the results.

**Table 4:** Correlations between PES-NWI dimensions and the satisfaction and Burnout variables (n=130). Barcelona, Spain, 2014-2015

Satisfaction	PES-NWI*		r Spearman^†^	r Pearson^‡^	
	Staffing and resources adequacy	Collegial nurse-physician relations	Nurses manager ability, leadership and support of nurse	Nursing foundations for the quality of care	Nurse participation in hospital affairs
Job satisfaction	0.013^†^	0.135^†^	0.058^†^	0.062^†^	0.139^†^
Hourly flexibility	-0.127^†^	0.128^†^	-0.079^†^	-0.013^†^	-0.125^†^
Opportunity	0.131^†^	0.096^†^	0.064^†^	0.034^†^	-0.117^†^
Autonomy	0.083^†^	0.159^†^	0.057^†^	0.048^†^	0.075^†^
Salary	-0.184^†^	0.041^†^	-0.125^†^	0.119^†^	0.049^†^
Training	0.144 ^†^	0.076^†^	0.077^†^	0.110^†^	0.095^†^
Vacation	0.341^†^	-0.103^†^	0.033^†^	-0.072^†^	-0.057^†^
Low Diseases	-0.069^†^	0.044^†^	-0.113^†^	0.035^†^	-0.133^†^
License studies	0.209^†^	-0.099^†^	0.072^†^	-0.111^†^	0.052^†^
*Burnout*					
Emotional exhaustion	-0.206^‡^	-0.230^‡^	-0.337^‡^	-0.249^‡^	-0.349^‡^
Depersonalization	-0.098^‡^	-0.205^‡^	-0.170^‡^	-0.202^‡^	-0.105^‡^
Personal achievement	-0.237^‡^	-0.266^‡^	-0.240^‡^	-0.355^‡^	-0.263^‡^

* PES-NWI = Practice Scale-Nursing Work Index;

† r = Spearman Correlation Coefficient;

‡ r = Pearson Correlation Coefficient

Finally, logistic regression analysis was performed to predict the intention of abandonment in dichotomous form (yes/no). Nurses who were dissatisfied with work are 2.3 times more likely to leave or change work than nurses who were satisfied. And nurses with emotional exhaustion also had 1.11 times more risk of intent to quit work than those who did not. The most positive perceptions of staff allocation and adequate resources were associated with a lower level of intention to abandon by nurses. Predictors that nurses intend to abandon or change work are shown in Table 5.

**Table 5:** Logistic regression: predictors of intention of perioperative nurses to abandon. n=130. Barcelona, Spain, 2014-2015

Variable Intention to abandon work				
	β*	SE^†^	p^‡^	OR^§^
**Emotional exhaustion**	**0.168**	**0.024**	**0.000**	**1.113**
**Dissatisfaction**	**0.894**	**0.225**	**0.000**	**2.304**
**Allocation of personnel and resources**	**-0.446**	**0.172**	**0.010**	**0.640**
**Constant**	**0.633**	**0.672**	**0.345**	**-**

* β = Estimated Parameter;

† SE = Standard Error;

‡ p = Chi-square;

§ OR risk

## Discussion

We sought to investigate the work environment of perioperative nurses of a high-tech public university hospital and its association with the work satisfaction, burnout and the perception of the quality of the care provided and the intention to abandon work. The PES-NWI scores for this study were not favorable. The means were lower than 2.5 for four of the five subscales of PES-NWI. The subscales of the dimension “Nursing fundamentals and quality care” presented higher mean score than those of “Nurse participation in hospital affairs”, which presented the lowest scores. Although these results point to the need for interventions to improve the general work environment in all areas, an analysis of the relation between the factors of work environment, satisfaction, burnout and intention to abandon work found significant associations. Moreover, 20% of nurses intended to quit work, having the work environment, work dissatisfaction and emotional exhaustion of perioperative nurses, predictive factors of this, which is consistent with similar studies^(^ 24 ^-^ 25 ^)^. Nurses who were dissatisfied with work are more likely to want to abandon or change work than nurses who were satisfied. And nurses with emotional exhaustion also intended to abandon, corroborating other studies conducted in other European countries^(^ 26 ^-^ 27 ^)^. In general, it was shown that, in this perioperative environment, more than half of the nurses were unsatisfied with their work and that, also, more than a third faced emotional exhaustion. There are studies in different countries with different health care systems that report similar deficiencies in the work environments and in the quality of care^(^ 2 ^,^ 25 ^)^. In the Spanish health system, the past economic crisis particularly affected the nursing profession by affecting the nurse/patient relationship, being a negative trend during these years and affecting the quality of care due to work overload^(^ 28 ^)^.

Other studies showed that the environments that were perceived by nurses as positive were translated into greater job satisfaction, lower levels of exhaustion and a lower intention of abandoning current employment and the profession, resulting in improved patient care^(^ 29 ^)^. In the current study, there is an association between the environment of perioperative nurse practice and the intention to abandon, especially a negative association with the size of staff and resources. According to recent studies, staff shortages result in a work overload with significant and direct effects on adverse events in patients^(^ 11 ^,^ 30 ^)^. And even if the dimensions of the work environment such as quality foundations and the employment relationship between nurses and physicians seem satisfactory, the central problems are in the design of work and the management of personnel that threaten quality care to the patient. It is crucial to solve these problems, which are susceptible to intervention of institutional management, to preserve the safety and quality of patient care. These results are consistent with the findings that have been described in other studies that were conducted by the European RN4CAST^(^ 10 ^,^ 31 ^)^.

The dimension that was best valued by perioperative nurses regards to the quality of care they provide, which was good and is comparable with other studies with better environments^(^ 32 ^)^. Perioperative nurses have a training of specialists or those graduated in perioperative care - although there is still no specialty course on perioperative nursing - which requires specialized skills from them^(^ 33 ^)^. The absence of a training system that is regulated for perioperative nursing staff in Spain will stand out as a limitation to solve by our health system. Moreover, nurse swindling decreases knowledge and experience in an organization. Therefore, if improvements directed to continued and advanced training are made, together with the professional development of nurses, the system can increase the satisfaction of nurses and contribute to them staying in their jobs and retaining the professionals. Studies in the literature often highlight the importance of education and the development of nurses to create a positive work environment^(^ 34 ^-^ 35 ^)^.

In this study, the environment was also characterized by good colleague relationships between nurses and physicians, although there is an important margin for improvement, similar to other similar studies^(^ 14 ^)^. In the studies of *Magnetic Hospitals*, the quality of collaboration between nurse and physician was an important feature of a healthy, high-performance work environment that increases the retention of nurse^(^ 15 ^)^. Furthermore, they reported few opportunities to participate in decision making in their unit. In another study, it was demonstrated that the participation of nurses in decisions improve efficiency and efficacy at the unit level^(^ 11 ^)^. Nurses’ dissatisfaction was mainly related to the few opportunities for professional development and wages. The economic remuneration they perceive is not in proportion to the work they provide and educational requirements. These aspects were also negative in the studies of RN4Cast Spain^(^ 10 ^)^, so it will reflect on the priorities in our Spanish Sanitary System and not only in our organization.

According to data from the Organization for Economic Cooperation and Development (OECD), the mean number of nurses in the European Union (EU) is 8.8 per thousand inhabitants and in Spain of 5.5. Our health system has almost the same number of nurses as doctors, whereas in the EU the number of nurses doubles that of doctors^(^ 36 ^)^. The WHO advocates for the need to have more nursing professionals to meet the needs of the population, although nursing care is considered necessary, effective and profitable. A report that was published by the World Nurse Innovation Summit (WISH 2018), in coordination with the Nursing Now campaign - which aims to empower the nurse profession - recommends investment in nursing and implement legislation , training and effective jobs to achieve the necessary improvements in health and health care. The need for a fundamental change in health policies, both globally and nationally, is indicated, which recognizes what nurses can achieve if they are allowed^(^ 37 ^)^. With regard to hospital management, the need for change to authentic leadership that is focused on professional growth and to foster a positive work environment can improve flows and workload, wages and resources for nurses^(^ 38 ^)^. Regarding comparisons of the perioperative environment or other environments, our study found that nurses perceive their work environment more unfavorable than nurses working in *Magnetic Hospitals*, following the same line as the rest of the Spanish hospitals^(^ 10 ^)^ and that of some European hospitals^(^ 25 ^)^. Different studies were conducted to evaluate the quality of work environments and compared their environments with *Magnetic Hospitals*. Although they obtained better results, they coincide that *Magnetic Hospitals* achieved better evaluations in the five dimensions of PES-NWI^(^ 39 ^-^ 40 ^)^.

Some researchers, who started the research of *Magnetic Hospitals*, explained that the same positive results can be achieved by introducing changes in the organization - it is not necessary to undertake the journey to magnetic status - such as inclusion of magnetic values in nursing work environments^(^ 41 ^)^. Moreover, we expose that, if magnetic values in hospital areas provided high quality standards, because they have a good relationship between the organizational model, the quality of care and patient safety, it is possible transferring it to our perioperative area.

Methodologically, our response rate was excellent and this can be attributed to the nurses’ commitment to the study and the support of managers. However, although the total population of nurses in the surgical area responded, i.e., a sample of 130 nurses in a single hospital, the results should be interpreted with caution. Our findings will allow management nurses to introduce improvements to increase the retention of perioperative nurses. Moreover, this study could be useful for other researchers in Spain and internationally to investigate perioperative environments. An important limitation of this study - given its cross-sectional nature - is that the nurses were asked about their intentions to change or abandon their work, and there was no follow-up in the following year to determine whether they actually abandoned their posts. Recently, a qualitative study was conducted by the same authors who reported on satisfaction, dissatisfaction and possible causes of work abandonment^(^ 42 ^)^. We recommend future studies with longitudinal designs or intervention drawings.

## Conclusion

In the perioperative care environment of this hospital, especially, the lack of personnel and resources, dissatisfaction and emotional exhaustion provoke, in perioperative nurses, the intention to abandon their work. One will consider implementing strategies for the retention of perioperative nurses, improving predicting factors such as: the work environment, especially the allocation of personnel and resources, dissatisfaction and emotional exhaustion. Creating positive work environments that are based on magnetic values can be a key strategy.
